# Rachel Challice's Vow

**Published:** 1888-01-07

**Authors:** 


### RACHEL CHALLICE'S VOW.

By the Author of "A Future on Trust," "Juno," &c.

### CHAPTER I.—THE VOW.

"Yet here for me, though heart and will are master

As strong as iron and as calm as death,

The will will waver, and the heart beat faster."

—*Herman Merivale.*

"RACHEL, child, are you there?"

"Yes, Granny, I am here. What do you want?"

"It is very dark; I can't see you. Get a candle, girl, and stir the fire. I've a deal to say, but I must have the light first to see you by. Make haste, to-morrow will be too late; I must say it to-night, or never."

The girl rose obediently from her place by the bedside. She had been sitting there, in the gathering gloom, almost motionless, for the last two hours; her eyes fixed on the still form lying beneath the bedclothes—a form so still that only the laboured breathing, or some feeble spasmodic movement, at times betokened life.

That life was ebbing quickly now, as Rachel instinctively felt. The last few grains in the hour-glass were slipping rapidly away. When the morning sun next rose, Rachel and Esther Challice would probably be alone in the world. Rachel stirred the smouldering embers in the grate, and laid on a few sticks, that quickly caught fire, making flickering flames throw dancing, quivering shadows over the little room. Then, reaching down a heavy brass candlestick from the mantelpiece, the girl placed a candle in it, which she lighted and put on the little table by her grandmother's bed. The solitary candle and the flickering fire-flames lit up a weird picture. They showed the white, drawn face of the old woman, who unwillingly was hurrying away to her sojourn in the vague land of spirits; who was fighting with all her feeble strength to retain a hold on the life Death was so inexorably drawing from her grasp, telling her meanwhile, mockingly, to remember that she had been granted a long respite—five years beyond the allotted threescore and ten—so that now there was no reasonable pretext for wishing to renew her lease of life.

Rachel had come back to her former place, and was sitting, motionless as ever, on the hard upright chair, her grey eyes still bent on the worn-out old figure in the bed.

Rachel Challice was no beauty—so, at least, people said. Casual observers did not see any special comeliness in the pale, oval-shaped face, the firm mouth, the grey eyes, with their long black lashes. She wanted life and expression, they said. They knew nothing of the habitual repression she had experienced from childhood; the harshness displayed towards her; the want of sympathy which had made her bury all signs of feeling deep down in her heart, so that chance observers saw only the smooth, unruffled surface. But a physiognomist, or clever discerner of character, would have read beneath that assumed coldness the real fire and energy of the girl's nature, would have guessed the unflinching determination, the passionate devotion, that underlay the crust of indifference. Her grandmother, who thought her a useful, dull sort of girl, had drilled, and snubbed, and repressed her in every possible way. She trusted, and in a vague way valued the girl; but she had never loved her.

All old Mrs. Challice's affection had been poured out in lavish abundance on her younger grandchild, Esther, a girl of nineteen, five years Rachel's junior; a lovely, bright, shallow-hearted thing, whose only god was pleasure, and whose first thought was for self.

Suddenly Mrs. Challice roused herself, and began to speak again.

"Come closer, Rachel; put your chair nearer the bed. I want to look into your face, and you must listen to every word I say."

"Yes, Granny," replied the girl, impassively, doing as she was bidden.

"I want to speak to you about Esther. She'll miss me sorely, will the dear child."

"Yes, Granny," again replied the elder granddaughter.

It was not an assent to the statement—more a sort of mechanical assurance that Rachel was attending to the subject on hand.

"She'll have no one to turn to when I'm gone," went on the old woman with a sort of quaver in her feeble voice, and a gleam of softness in the hard expression of her steely, still bright eyes.

"You'll look after her and mind her, I know that; that isn't what I meant. But she wants love, does my little Esther, and you aren't the sort of girl to show or feel that, you know, Rachel."

"Yes, Granny," came again the same stereotyped assent.

"It's that thought that holds me back to life," pursued Mrs. Challice impatiently, while she turned her head restlessly on the pillow, as though chafing at her inability to successfully grapple with and rout Death. "But, Rachel, I've alway depended on you. I know that when you promise a thing you'll do it, even if it costs you a struggle. So now I want you to swear to do something I ask you. It'll be hard work, with your cold, stiff nature; but if you'll promise I know you'll do your best, and I can't die happy till I've heard you say it."

A faint shadow of impatience passed over the girl's face, a slight contraction of the smooth forehead, a quiver of the firm mouth. But it was all so slight, so instantaneous, that it might easily have been attributed to some reflection of the flickering, deceptive light. A moment later, and her countenance had regained its usual look of impassive serenity.

"Tell me what you want me to promise."

"It's Esther, Rachel. My poor, pretty, little darling! She'll miss her old granny so, Rachel. You don't love her as I do; you haven't got it in your nature."—Again the quivering, momentary look of scorn on the girl's placid face. —"She can't stand alone," went on the old woman. "She is too pretty, too soft and loving. It would kill her to meet with nothing but hardness, and the world is hard enough towards such little helpless things as my Esther. You are strong, Rachel; hard words and looks don't hurt you. Promise me, child—swear to me that you'll always stand between Esther and any harm; that you'll think of her first and yourself last; that you'll make life as sweet as you can for my little one at any cost to yourself."

It was a cruel promise to extort. Rachel Challice felt it so. There was no thought for her. Nothing was to be done for her good. In death—as in life—all Mrs. Challice's thoughts were centred on Esther—on the grandchild who had done the least, and yet who was so unjustly loved the best.

"Swear it, Rachel!" she went on, excitedly. "Don't be so hard! Can't you understand that it is your promise only that can let me die easy? Can't you see reason in all I say? Promise to guard Esther! Give yourself up body and soul for her good! It oughtn't to be such a hard thing for an elder sister to do."

Rachel Challice loved her sister passionately. She was ready enough, of her own free-will, to perform all—and a hundred times more than the duties dictated by her grandmother.

It is true she never displayed her absorbing love for the pretty, silly little sister by kisses, or loving words, or any of the ordinary ways of showing affection. Her pent-up, sensitive nature shrank from any, even the most trivial, tokens of what she termed sentimentality.

So both Esther and her grandmother had grown to look upon Rachel as odd, cold, and heartless.

But the more she was misjudged, and even to her face rebuked for her apparent indifference, the more firmly did her secret passionate affection for her little sister increase. She was such a fanatic in the matter, that she felt her love would be desecrated and spoiled were she to display it openly, or by words own to its existence. It was to her mind too fragile and precious a treasure to drag forward to the coarse gaze of common observation and inquiry.

So it lived—unrevealed and undisplayed—shut up in the dark recesses of Rachel's heart; there fructifying and increasing, as do the bare unlovely seeds in the bosom of mother earth, waiting for harvest time to come, and the full wealth of luxuriance to burst upon the glad eyes of the astonished ingatherer.

Such being her feelings for Esther, Rachel was deeply wounded at the bare idea of being made to promise protection to her in the future. She considered such a line of action a matter of course—a thing that, without any suggestion from a third person or verbal declaration from herself, would naturally come to pass.

She forgot that others could not see into her heart; that her habitual assumed passiveness had gained her the credit of indifference; that her grandmother had no guarantee for believing in her never-displayed affection for her sister. How could Mrs. Challice know by intuition that her granddaughter had already secretly vowed to pursue the course to which she was now urging her? Rachel, in her pride and repression, was unreasonable.

She could have brought forward many extenuating circumstances had she chosen, poor girl! But she had grown so accustomed to hear herself misjudged that she had ceased to care to be understood, and consoled herself with the cold comfort that she herself knew better; that she was not the heartless monster her grandmother always, and her sister on occasions, chose to represent her.

"Rachel, Rachel, promise me!" urged the quavering voice from the bed. "Speak, girl, don't sit there like a stone. Isn't your own flesh and blood worth giving a thought for at least?"

"I was thinking, Granny," replied the girl in her even, monotonous voice. "Yes, I will promise to do what you wish if it will make you happier."

"Swear it, girl! Don't speak like that, as if Esther's whole future did not lie with you! Here, get the Bible, take it in your hands, and say it after me."

The girl's cheeks grew paler, and her lips were pressed more tightly together. It was all she could do to restrain her feelings, though long practice had made her a past mistress in the art.

"I, Rachel Challice, swear to protect my sister at any cost to myself. To shield her, support her, work for her, and never leave her so long as I can help her—So help me God!"

Mechanically, sentence by sentence, she repeated the words dictated by the old woman. Then quietly, as though she had been rehearsing the most trivial lesson, she laid down the book, and said, "It is time for your beef tea now. Lie down, Granny; I will bring it to you at once."

"Heartless girl!" moaned the old woman, as her grandchild left the room. "My poor, tender Esther! How will she bear it! But Rachel has promised, and I've never yet known her break her word. She will shield Esther, if she does not care for her; and my bonny pet will find plenty to love her, with her sweet ways and pretty face. There's Harry Evans and David Stephens, they're both after her. Aye, I'd like her to marry David; a good, steady lad he is, and one who'll make his own way in the world."

Night drew swiftly on.

It was October, and the purple heather and green bracken were withering on the sides of the mountains. There was a white patch on the summit of Snowdon, herald of the coming frost-bound winter. The sun sank down early behind Siabod peak, and chill winds swept along the valleys of the Colwyn and the Glasslyn.

Mrs. Challice had lived in the cottage just standing back from the road that runs from Beth-gelert to Ryhd-dhu, for the last five years, ever since her husband, Lord Merthyn's gamekeeper, died, leaving his wife and their orphaned grandchildren to live on the pension his master, for the sake of John Challice's long and faithful services, allowed them. They were the children of a daughter whose sad history need not be told here, and who had long years ago been laid to rest in the quiet churchyard of Beth-gelert.

From the day of her birth old Mrs. Challice had looked coldly on her elder grandchild. The girl knew her grandparents disliked her. From her earliest childhood she had instinctively grasped the fact, and had schooled herself to her present state of morbid self-restraint. While Esther would play tricks on her grandfather, sit on his knee, lay her soft, rosy cheeks coaxingly against his wrinkled brown face, or tease and hinder her grandmother like a frisky kitten, Rachel would soberly and steadily be fulfilling the daily household tasks allotted to her, only speaking when poken to, and then returning the shortest answers possible.

"She's got no heart, hasn't the child," old John would observe, taking his pipe out of his mouth for a moment, and shaking the ashes into the grate; while Mrs. Challice would reply bitterly, "She's none of us. Rachel is her father's girl, and will never be a blessing to nobody."

The old couple were not careful in their remarks. They commented freely on the difference between the children in the very presence of the girls themselves. Thus Rachel's hardness was fostered, and Esther learnt to despise and condemn her sister, though in a selfish, shallow way, she loved and depended on her as well.

She was a bright, winsome little being, Esther Challice, the living image of her dead mother. She had the same laughing, blue eyes, the same weak-shaped, rosy-lipped mouth, the peach-tinted complexion, and curling golden hair that had belonged to the elder Rachel Challice in her youth.

As the grandparents watched Esther, their minds would travel back, and they would fancy that they saw before them once again their only child, in the full beauty of youth and innocence, as she had been before sorrow had aged and weakened her, and disfigured and spoiled her fresh girlish loveliness.

She was gone now; sleeping beneath the dark shadow of the massive yew trees, within sound of the brawling Colwyn and the rippling Glasslyn rivers, that meet beneath the old bridge leading to the sombre Welsh graveyard. But in Esther she had left as a legacy her living image. And on her the old people poured out the lavish store of love they had once expended on their daughter.

As in past days, they could see no fault in the giddy girl who wheedled and coaxed them, teased and worried them, and made them laugh in spite of themselves; whose tears came so readily at the slightest word of reproof, and dried as quickly as the ready forgiveness was accorded.

Experience had taught them no lesson. They were as ready as ever to believe in the glitter of the sham gold, and to close their ears to the false ring of its base metal.

Eight o'clock had struck, and Rachel still sat watching the quiet face of the dying woman.

The white moon shone down on the rugged mountains outside. Hebog was bathed in a sea of silver radiance, his bare, rough-hewn outline standing softened and mist-veiled against the dark blue of the night sky. The peak of Arran was crowned with the same lustrous glamour. Colwyn rushed, foam-flecked and brawling, over the stones and rocky boulders that strew its bed; and, beyond, the dreary tract of moorland shelved upwards till it reached the base of the mountains, and lost its outline in the deep purple shadows cast by the mighty hill monarchs.

"Where's Esther? Where's my little one?" moaned the old woman.

Her voice had grown perceptibly weaker since she had last spoken. As Rachel looked closer, a film seemed spreading over the blue steely eyes, and there was a drawn livid look on the face that had not been there half-an-hour before.

The shadow of the mighty enemy of mankind was reflected on the countenance of Mrs. Challice. Death had stalked into that quiet, half-lit room, had taken the old woman by the hand, and was peremptorily bidding her follow him. And she, loth and resisting, was struggling powerless in the strong grip of the remorseless tyrant.

"Esther is at the Evans', Granny," replied Rachel. "You know she went there to tea and to spend the evening, and won't be back for an hour or more."

"Aye, best so—best so," muttered the old woman, feebly. "But I'd have liked to have seen her first. Tell her, Rachel—Rachel——."

The faint voice grew weaker and more broken, the eyes glazed and staring, the death-rattle sounded in the old woman's throat, while Rachel knelt helpless and trembling by the bedside.

Then all was still.

The white moon shone brilliantly outside, and peeped in between the parted folds of the window curtains.

The flickering fire flames and the solitary flaring candle illumined with fitful gleams the tiny, dingy bed-room.

The stars trembled in the blue evening sky, and were mirrored on the glassy bosom of Dina's lake.

White moon, red fire flames, and silver stars shone on the ghastly face of a dead woman, and lit up the scarcely less pallid, awe-stricken countenance of Rachel Challice, as, in the shadow of the darkness of death, she knelt rigid and motionless by the lifeless body of her grandmother.

*(To be continued.)*

